# Bp-Bs, a Novel T-cell Engaging Bispecific Antibody with Biparatopic Her2 Binding, Has Potent Anti-tumor Activities

**DOI:** 10.1016/j.omto.2019.03.009

**Published:** 2019-04-02

**Authors:** Jiayu Liu, Xiaoqiong Wu, Limin Lin, Haitao Pan, Yanlan Wang, Yumei Li, Yining Zhao, Zhong Wang

**Affiliations:** 1 School of Pharmaceutical Sciences, Sun Yat-Sen University, Guangzhou 510006, China

**Keywords:** Her2, bispecific, biparatopic, bivalent, single domain, antibody, trastuzumab

## Abstract

Patients with Human epidermal growth factor receptor type 2 (Her2) overexpression are associated with aggressive tumor growth and poor clinical outcomes. Bispecific antibodies targeting Her2 have recently exhibited potent effects on Her2 signal inhibition. In this study, a novel biparatopic anti-Her2 bispecific antibody (Bp-Bs) was constructed by linking a single anti-CD3 Fab with two different anti-Her2 single-domain antibodies targeting non-overlapping epitopes of Her2. The Bp-Bs demonstrated strong binding on Her2-positive cells and potent cytotoxicity on Her2-positive tumor cells, even Her2-low expression cells, suggesting that biparatopic bispecific antibodies may have improved therapeutic benefits on broad Her2 patient populations.

## Introduction

Human epidermal growth factor receptor type 2 (Her2, also named Her2/neu or ErbB2) is a member of the HER family of transmembrane receptor tyrosine kinases.^1^ It comprises a cytoplasmic tyrosine kinase region, a single membrane-spanning region, and an extracellular region of about 630 amino acids that contains four different structural domains (domains I–IV). The HER2 oncogene is overexpressed and functionally important in 25%–30% of human primary breast tumors^2^ and in various other human cancers, such as lung, gastric, oral, and colorectal cancers.^3^, ^4^, ^5^, ^6^, ^7^, ^8^

The vital role of Her2 in breast cancer development prompted therapeutic development targeting Her2. The development of the anti-Her2 therapeutics trastuzumab, lapatinib, pertuzumab, and T-DM1 has achieved clinical benefits for Her2-positive patients.^9^ However, low response rates and resistance are also associated with current therapeutics. For example, only 15%–30% of Her2-positive patients respond to trastuzumab therapy due to *de novo* and acquired resistance.^10^, ^11^ Trastuzumab has minimal effects on Her2 low- or medium-expression cancer cells *in vivo* and *in vitro*.^12^, ^13^ Poor internalization also leads to resistance in T-DM1 treatment of metastatic breast cancer.^10^, ^14^, ^15^ Thus, new therapeutic options are urgently needed for patients resistant to or with no response to current Her2-targeted therapies.

To improve the efficacy of Her2-directed antibodies, a growing list of novel formats targeting Her2 have been proposed, including combination therapy and bispecific antibodies. As trastuzumab and pertuzumab bind to non-overlapping epitopes on Her2, the combinatorial treatment of these two antibodies provided more clinical benefits than either antibody alone (CLEOPATRA trial^16^). Trastuzumab combined with pertuzumab plus docetaxel has already been approved for the first-line treatment of patients with Her2-positive metastatic breast cancer.^17^ Bispecific antibodies targeting both Her2 and Her3,^18^ targeting two different epitopes of Her2,^19^, ^20^ or engaging T cells to Her2 cancer cells by targeting Her2 and CD3^21^ have also exhibited encouraging results in phase I or pre-clinical studies.

In previous studies, we have designed bispecific antibodies using anti-Her2 single-domain antibodies (or VH-only heavy-chain antibody [VHH]) that redirect immune cells toward Her2 cancer cells.^13^, ^22^, ^23^, ^24^ These bispecific antibodies exhibited potent cytotoxicity against Her2 medium- or high-expression cells.^12^, ^22^ However, these antibodies still had no or low cytotoxicity against Her2 low-expression cells, such as MCF7 cells.^12^, ^22^ As monovalent anti-Her2 was used in these formats, we reasoned that, by increasing the valency of the anti-Her2 module, the binding and cytotoxicity of the bispecific antibody may be enhanced. Furthermore, the biparatopic antibody, by engaging two different epitopes of Her2, has shown enhanced anti-tumor activities.^25^ Thus, we constructed bispecific antibodies employing two anti-Her2 VHH that target the same or two different epitopes of Her2 in this study. The Fab-based bispecific antibody (biparatopic anti-Her2 bispecific antibody [Bp-Bs]) exhibited potent cytotoxic activities against Her2-positive cells, even Her2 low-expression cells, suggesting biparatopic bispecific antibodies may be beneficial to broad patient populations, including Her2 low-expression cell patients.

## Results

### Construction, Expression, and Purification of Bispecific Antibodies

**Figure 1:**
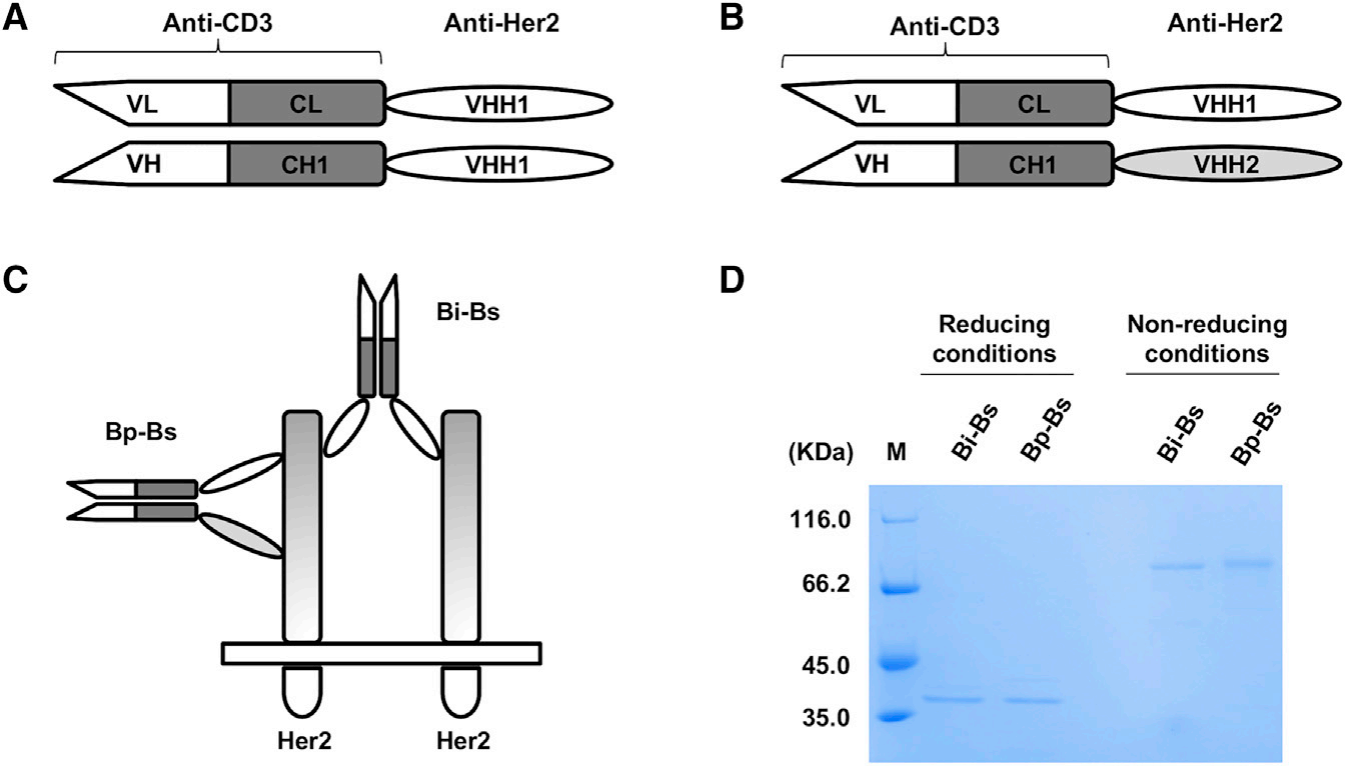
Design and Purification of Bi-Bs and Bp-Bs (A) Structure of the bispecific antibody (Bi-Bs), which was formed by the heterodimerization of VL-CL and VH-CH1 of humanized anti-CD3 antibody (UCHT1 clone) linked with an anti-Her2 VHH1 to the C terminus of CL or CH1. (B) Structure of the biparatopic bispecific antibody (Bp-Bs). Different from Bi-Bs, another anti-Her2 VHH2 was linked to the CH1. (C) Schematical representation of Her2 recognized by Bi-Bs and Bp-Bs. (D) Coomassie blue staining of purified Bi-Bs and Bp-Bs after immobilized Ni-NTA affinity chromatography at reducing or non-reducing conditions. M, molecular weight markers.

To design more potent anti-Her2 molecules, bispecific antibodies, including bivalent anti-Her2 bispecific antibody (Bi-Bs) and Bp-Bs, were constructed (Figures 1A and 1B) by linking single-domain anti-Her2 VHH1 or anti-Her2 VHH2 to the carboxyl-terminal of the CH1 or CL domain of an anti-CD3 antibody (UCHT1). They were then cloned into *E. coli* expression vectors pET26b and pET21a, respectively. The pelB signal peptide was added to the N termini of the two constructs for periplasmic expression and secretion in *E. coli*. The Bp-Bs was formed via the heterodimerization of VH-CH1-VHH2 and VL-CL-VHH1, as anti-Her2 VHH1 and anti-Her2 VHH2 bind to different epitopes on Her2 protein (Figure 1B). The Bi-Bs was formed via the heterodimerization of VH-CH1-VHH1 and VL-CL-VHH1 (Figure 1A). The binding of Bi-Bs and Bp-Bs on Her2 protein is shown in Figure 1C. The antibodies were induced and expressed in M9 medium.^26^, ^27^ The proteins were then purified by Ni-NTA agarose affinity chromatography and analyzed by SDS-PAGE. The relative mobilities of the purified proteins on SDS-PAGE were consistent with the expected molecular masses of about 39 kDa for the individual chains of Bi-Bs or Bp-Bs under reducing conditions and 78 kDa for Bi-Bs or Bp-Bs under non-reducing conditions (Figure 1D).

### Binding Characteristics of Bispecific Antibodies

**Figure 2:**
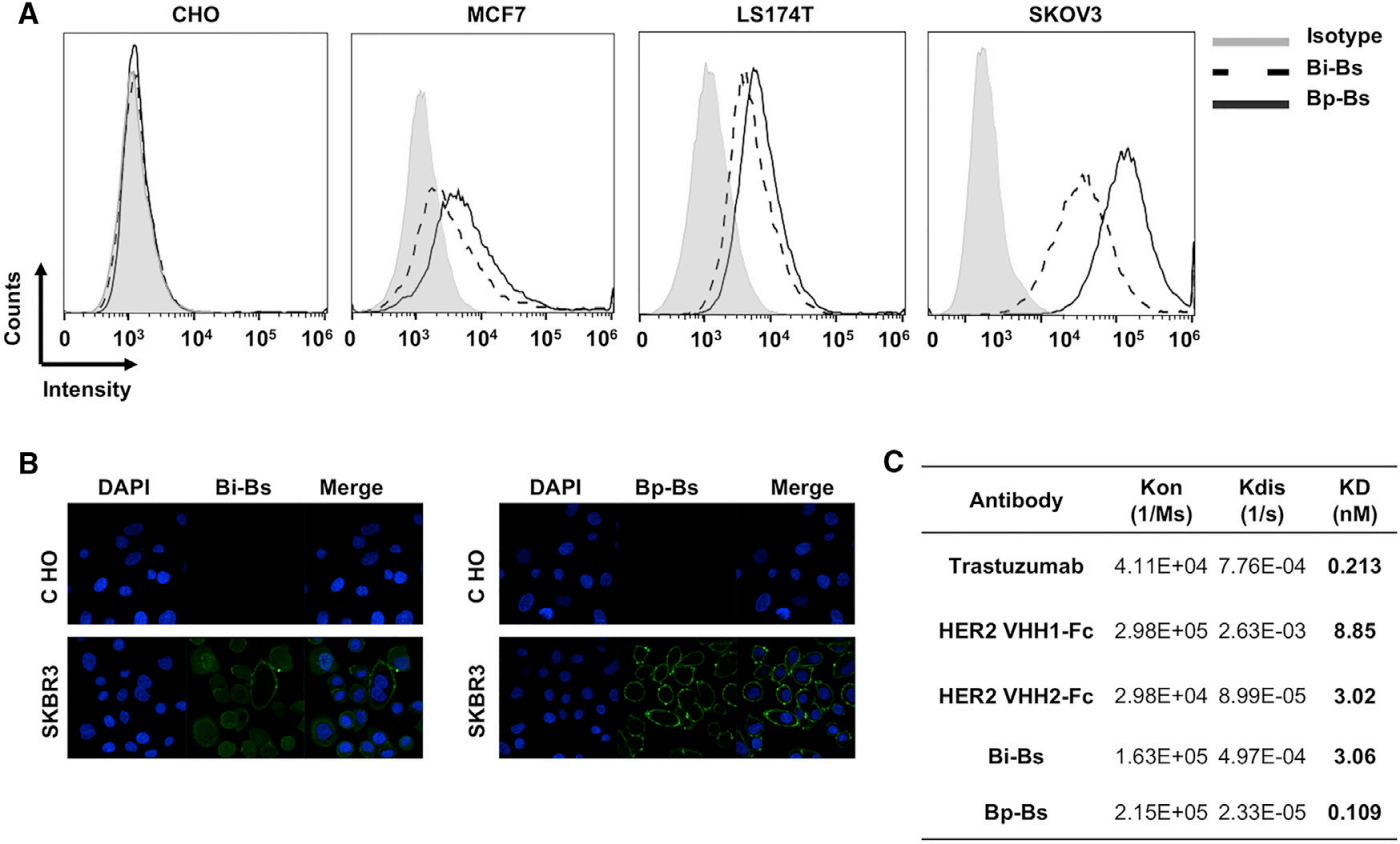
Bp-Bs Can Bind Her2 with Higher Affinity (A) Flow cytometry analysis of Bi-Bs and Bp-Bs on Her2-negative CHO cells, Her2-positive LS174T and SKOV3 cells, as well as Her2 low-expression MCF7 cells. (B) Confocal microscopy of immunofluorescence staining of Bi-Bs (left) and Bp-Bs (right) on CHO cells (top) and SKBR3 cells (bottom). (C) Binding affinity measurement results of Bi-Bs and Bp-Bs to Her2 antigen.

To assess the binding of the bispecific antibodies to Her2, flow cytometry was employed using cell lines, including Her2-negative cell line Chinese hamster ovary (CHO), Her2-positive cell lines LS174T and SKOV3, as well as Her2 low-expression cell line MCF7. Cells were incubated with purified bispecific antibodies, and then they were detected with AF488-conjugated anti-human immunoglobulin G (IgG) (H+L) antibody. No binding of bispecific antibodies to CHO cells was observed (Figure 2A). Specific binding of the Bi-Bs or Bp-Bs to the Her2-expressing cells was detected (Figure 2A). Bp-Bs also showed consistently increased intensity compared to Bi-Bs, suggesting enhanced binding of Bp-Bs to Her2-positive cells. Immunofluorescence analysis (Figure 2B) further confirmed the specific binding of Bi-Bs and Bp-Bs to the Her2-positive cell line SKBR-3, but not to CHO cells.

To further evaluate the binding of bispecific antibodies to Her2, the affinity of different antibodies to Her2 was measured. As controls, trastuzumab, anti-Her2-VHH1-Fc, and anti-Her2-VHH2-Fc have the affinities of 0.213, 8.85, and 3.02 nM, respectively (Figure 2C). The Bi-Bs, which is based on anti-Her2-VHH1, showed a similar affinity as the anti-Her2-VHH1-Fc (Figure 2C). Bp-Bs showed increased affinity (0.109 nM) compared to Bi-Bs (3.06 nM) (Figure 2C), consistent with increased binding by flow cytometry analysis (Figure 2A), suggesting that Bp-Bs binds to Her2 with higher affinity than monoparatopic bivalent antibodies.

### T Cell-Mediated Cytotoxicity Induced by Bispecific Antibodies

**Figure 3:**
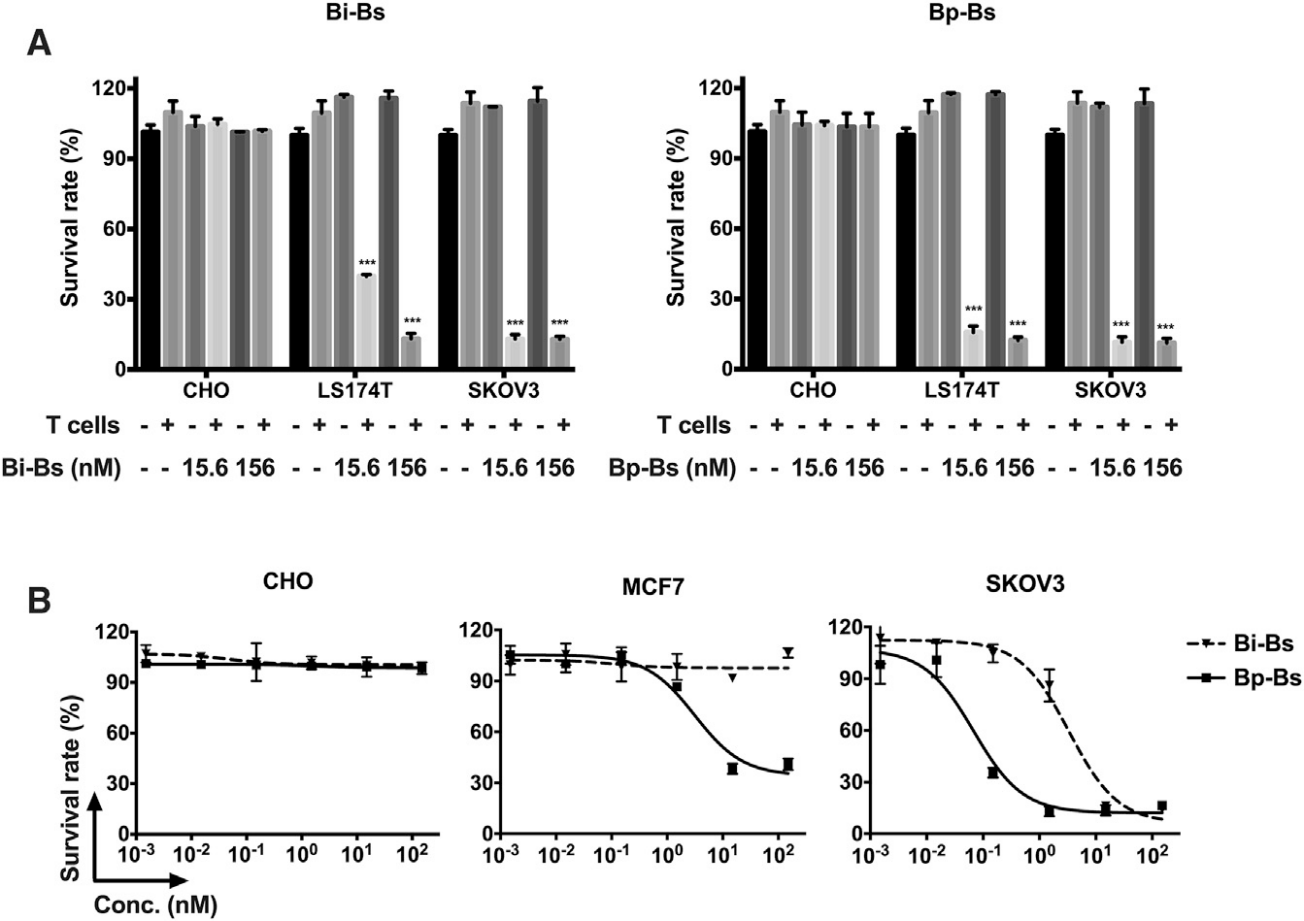
Bi-Bs and Bp-Bs Induced T Cell-Mediated Cytotoxicity (A) The cytotoxic activities of Bi-Bs and Bp-Bs were analyzed as described in the Materials and Methods. Different cell lines were treated with the indicated concentrations of antibodies with or without fresh isolated T cells. (B) Dose-dependent cytotoxicity assays were performed with human CD3^+^ T cells as effector cells and CHO, MCF7, and SKOV3 as target cells (effector-to-target ratio [E:T] = 10:1), in the presence of the indicated antibodies. The concentrations of antibodies were from 150 to 0.0015 nM. The mixtures were incubated for 72 h before the cytotoxicity measurement. All the data are the mean of triplicates, with the error bars representing the SD (***p < 0.001 versus tumor cell + T cells, Dunnett’s multiple comparisons test).

The cytotoxicities of Bi-Bs and Bp-Bs were then evaluated using Her2-negative CHO cells and Her2-positive cell lines LS174T and SKOV3. No cytotoxicy was observed for Her2-negative cells, even in the presence of T cells for both bispecific antibodies (Figure 3A). In the absence of T cells, neither Bi-Bs nor Bp-Bs inhibited tumor cell growth for Her2-positive cells (Figure 3A). With the presence of T cells, the antibodies at either 15.6 or 156 nM can decrease the viability of Her2-positive cells LS174T and SKOV3 (Figure 3A). As trastuzumab and monovalent anti-Her2 bispecific antibodies had no or low cytotoxicity against Her2 low-expression cells, such as MCF7 cells,^12^, ^22^ the cytotoxic activities of Bi-Bs and Bp-Bs were tested using MCF cells (Figure 3B); Bi-Bs had minimal cytotoxicity against MCF cells, while Bp-Bs showed cytotoxicity against MCF cells (Figure 3B). Moreover, Bp-Bs also showed more effective cytotoxicity than Bi-Bs in the Her2 high-expression cell line SKOV3 (Figure 3B), suggesting that Bp-Bs may provide enhanced cytotoxicity toward Her2 expression, even Her2 low-expression tumors.

### Her2-Signaling Regulation Induced by Bispecific Antibodies

**Figure 4:**
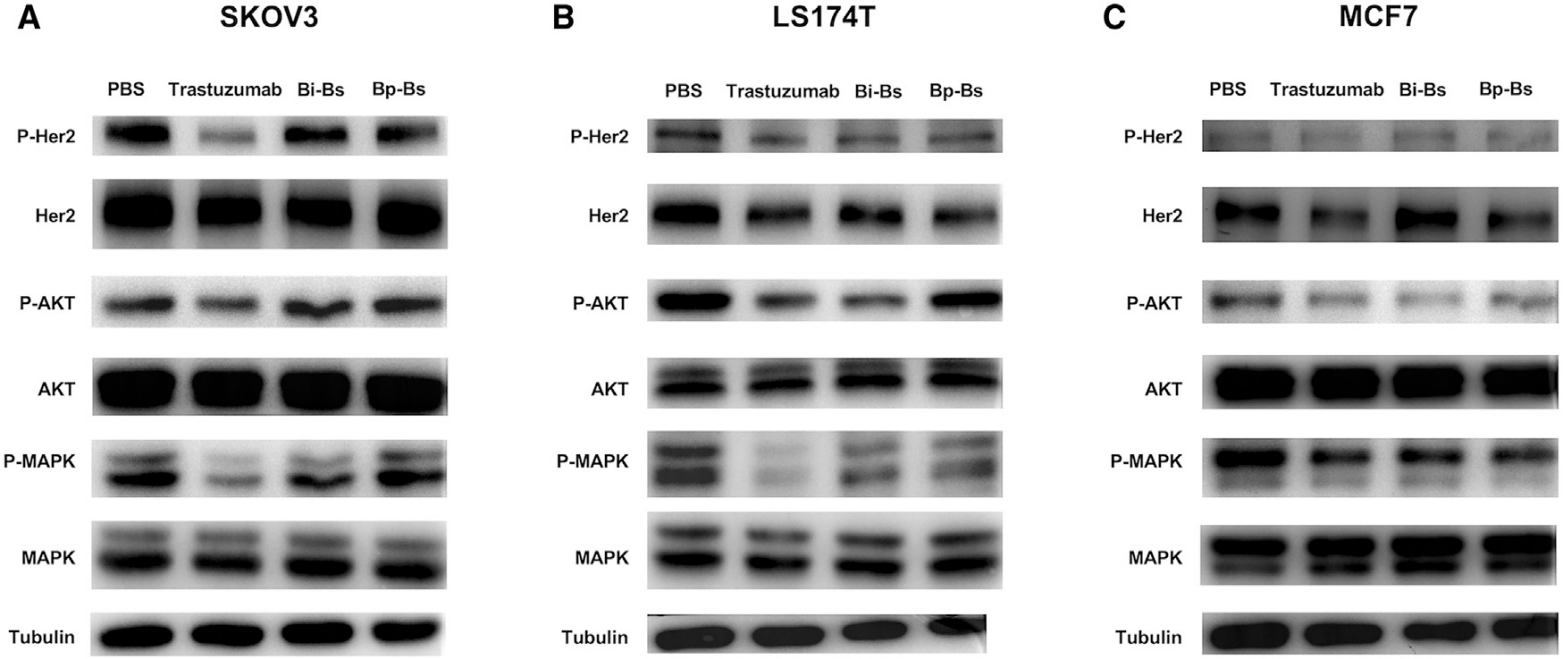
Bp-Bs Inhibits HER2 Downstream Signaling Different cell lines were treated with 100 nM of the indicated antibodies for 30 h. Total cell lysates were analyzed by immunoblot for the specified proteins or phosphorylated proteins. (A) SKOV3. (B) LS174T. (C) MCF7.

The effects of bispecific antibody treatment on the activation of Her2 and downstream AKT- and mitogen-activated protein kinase (MAPK)-signaling pathways were then evaluated using Her2-positive cell lines LS174T and SKOV3, as well as Her2 low-expression cell line MCF7. Inhibition of Her2 and the downstream PI3K pathway is important for the function of trastuzumab.^28^, ^29^, ^30^, ^31^, ^32^ Indeed, in LS174T and MCF7 cells, trastuzumab induced a decrease in Her2, MAPK, and AKT phosphorylation (Figure 4). Different from trastuzumab, both Bi-Bs and Bp-Bs showed minimal effects on the phosphorylation of Her2 and MAPK in all three cell lines (Figure 4). These results suggest that both Bi-Bs and Bp-Bs have minimal effects on the Her2-signaling pathways.

### Pharmacokinetic Analysis of Bispecific Antibodies

**Figure 5:**
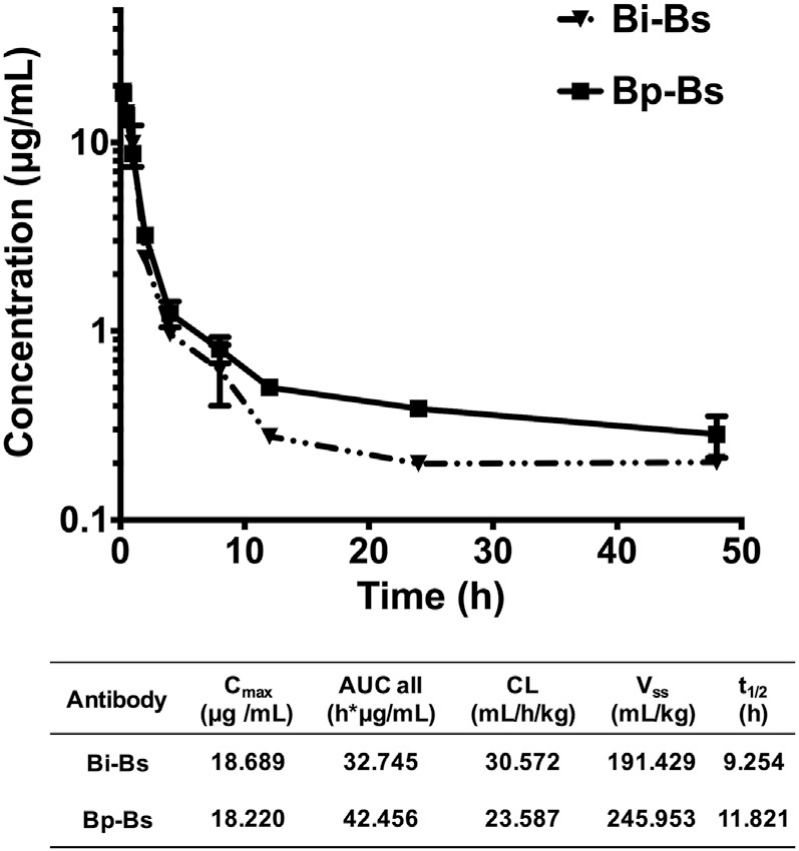
PK Profiles of Bi-Bs and Bp-Bs in Mice The serum concentrations of Bi-Bs and Bp-Bs were measured, as described in the Materials and Methods, after intravenous delivery in CB-17 SCID mice. The average of triplicates is shown with bars representing SD. The pharmacokinetic parameters based on the study are shown in the table (bottom). C_max_, maximal circulating concentration; AUC all, total area under the curve; CL, total clearance; V_ss_, the apparent volume of the plasma compartment; t_1/2_, half-life.

To perform *in vivo* efficacy studies, the pharmacokinetics of Bi-Bs and Bp-Bs were first evaluated in mice. A single dose of Bi-Bs or Bp-Bs was delivered intravenously to CB-17 severe combined immunodeficiency (SCID) mice. The antibody concentrations in the serum were then determined by ELISA. As shown in Figure 5, though Bp-Bs showed a slightly higher residue concentration after 10 h post-dosing, similar *in vivo* half-lives of Bi-Bs and Bp-Bs were observed (9.254 and 11.821 h, respectively).

### *In Vivo* Tumor Suppression Induced by Bispecific Antibody

**Figure 6:**
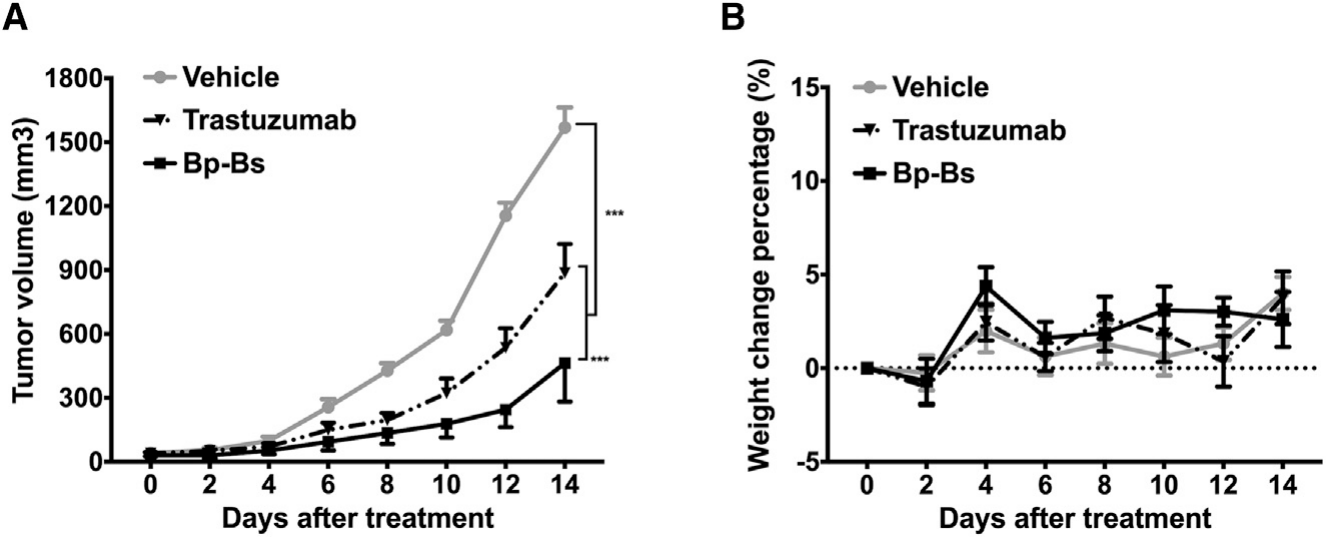
Bp-Bs Inhibited Tumor Growth *In Vivo* (A) NOD/SCID mice (n = 6 per group) were engrafted subcutaneously with LS174T cells (1 × 10^6^ per mouse). When the tumor grew to 50–100 mm^3^, the mice were then grafted with freshly isolated human PBMCs (5 × 10^6^/mouse) and treated intraperitoneally with vehicle (PBS, solid circle), trastuzumab (2 mg/kg, solid triangle down), or Bp-Bs (1 mg/kg, solid square) every 2 days. The tumor volume was then measured. The data represent the average tumor volume of six mice. The error bars represent the SE (***p < 0.001, Dunnett’s multiple comparisons test, vehicle versus trastuzumab and vehicle versus Bp-Bs; ***p < 0.001, paired t test, trastuzumab versus Bp-Bs). (B) The body weight changes of animals in different treatment groups. The data shown are the average percentage of weight changes with error bars representing the SE.

The potent *in vitro* cytotoxicity of Bp-Bs prompted us to examine whether Bp-Bs can inhibit tumor growth *in vivo*. Adoptive transfer models were performed using LS174T cells, which have low-to-medium Her2 expression. The cancer cells were engrafted subcutaneously into the non-obese diabetic (NOD)/SCID mice. After the tumor volume reached 50–100 mm^3^, the mice were grafted with freshly isolated human peripheral blood mononuclear cells (PBMCs). The mice were then treated with vehicle control PBS, trastuzumab (2 mg/kg), or Bp-Bs (1 mg/kg) every 2 days over the following 10 days. Significant anti-tumor activity was observed (Figure 6A), suggesting that Bp-Bs can suppress tumor growth *in vivo*. Moreover, Bp-Bs exhibited higher tumor inhibition activity than trastuzumab (Figure 6A). No body weight differences were observed for the different groups (Figure 6B).

**Figure 7:**
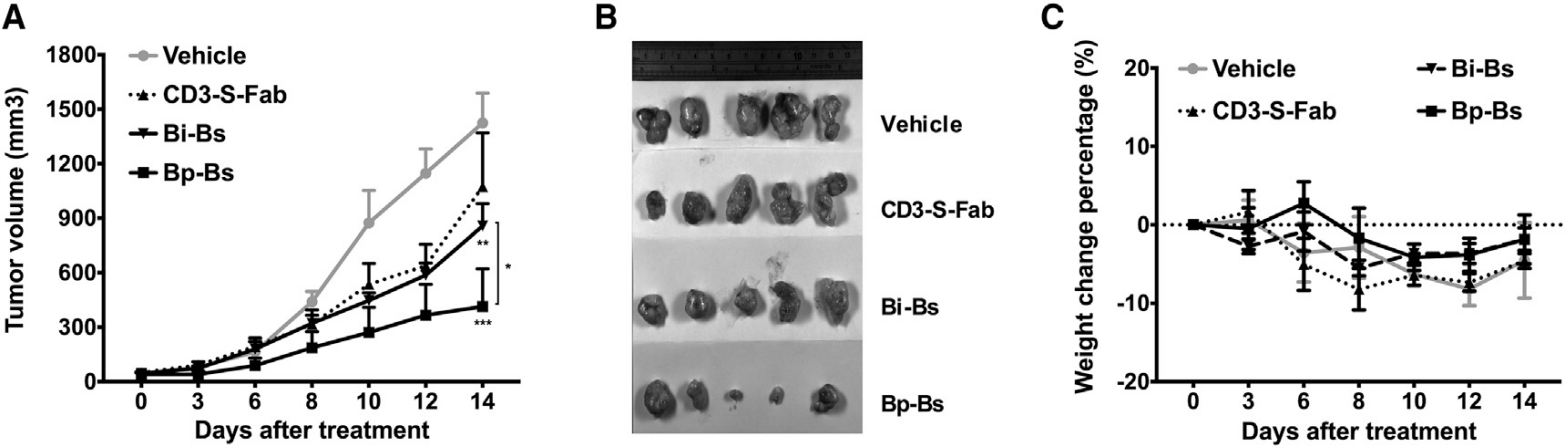
Bp-Bs Showed Stronger Tumor Suppression Activity Than Bi-Bs *In Vivo* (A) NOD/SCID mice (n = 5 per group) with subcutaneously engrafted LS174T cells were grafted with freshly isolated human PBMCs (5 × 10^6^/mouse) and treated intraperitoneally with vehicle (PBS, solid circle), CD3-S-Fab (1.5 mg/kg, solid triangle up), Bi-Bs (1.5 mg/kg, solid triangle down), or Bp-Bs (1.5 mg/kg, solid square) every 3 days. The tumor volume was then measured. The data represent the average tumor volume of five mice. The error bars represent the SE (**p < 0.01, vehicle versus Bi-Bs; ***p < 0.001, vehicle versus Bp-Bs; Dunnett’s multiple comparisons test; *p < 0.05, paired t test, Bi-Bs versus Bp-Bs). (B) Picture of tumors at day 14 after treatment. (C) The body weight changes of animals in different treatment groups. The data shown are the average percentage of weight changes with error bars representing the SE.

Previously, we reported a bispecific CD3-S-Fab that was constructed by linking an anti-Her2 VHH (Her2 VHH1) to the conventional anti-CD3 Fab (UCHT1) at the C terminus of VL-CL. CD3-S-Fab can be used for the redirection of T cells toward Her2-positive tumor cells and to kill Her2-positive cancer cells *in vitro* and *in vivo*.^13^ However, the monovalent binding to Her2 of CD3-S-Fab may not be efficient as a therapeutic agent. To investigate whether Bp-Bs also has a more potent anti-tumor activity than Bi-Bs or monovalent anti-Her2 bispecific antibody CD3-S-Fab,^13^ the mice were treated with Bp-Bs, Bi-Bs, or CD3-S-Fab at 1.5 mg/kg every 3 days. Tumor growth inhibition was then measured (Figure 7A). Similar to the *in vitro* findings, Bp-Bs showed much higher anti-tumor activities than monovalent CD3-S-Fab or Bi-Bs, while Bi-Bs had only slightly higher anti-tumor activity than monovalent bispecific antibody CD3-S-Fab (Figure 7A). Moreover, two of five mice had minimal tumor remaining after Bp-Bs treatment (Figure 7B). No body weight differences were observed for the different groups (Figure 7C). These data suggested that Bp-Bs has potent *in vivo* anti-tumor activities without gross toxicities in xenograft studies.

## Discussion

In this study, bispecific antibodies targeting Her2, Bi-Bs and Bp-Bs, were constructed and evaluated for their anti-tumor activities. Both bispecific antibodies exhibited potent *in vitro* and *in vivo* anti-tumor activities. Moreover, the Bp-Bs, by engaging two different epitopes of Her2, showed enhanced activities against Her2 cancer cells *in vitro* and *in vivo*. Bp-Bs even exhibited cytotoxic activities against Her2 low-expression cells. As downregulation of Her2 target expression is the primary mechanism of acquired resistance to T-DM1,^33^, ^34^ our study suggested biparatopic bispecific antibodies may benefit more broad patient populations. It may be used to treat patients with low Her2 expression and reduce relapse by eliminating cancer cells even with decreased Her2 expression.

In this study, the Fab format rather than common IgG was used to construct Bp-Bs. The Fab format can reduce the complexity of making bispecific antibodies, as no pairing of light chain is necessary. The low molecular weight of Fab and single-domain antibodies (around 13–15 kDa) can also reduce the overall molecular weight of a bispecific antibody, which may enhance tissue penetration and epitope access sterically. Moreover, the molecular weight of approximately 80 kDa, which exceeds the first-pass renal clearance threshold (∼50 kDa), may also offer a longer half-life and, hence, a pharmacokinetic advantage compared to smaller antibody constructs.

Although there are multiple promising formats of T cell-targeting bispecific antibodies under active pre-clinical and clinical development, only the tandem single-chain variable fragment (scFv; BiTE) and Triomab trifunctional format have been approved for clinical use.^35^ Unpredictable expression yields and spontaneous aggregation are the major difficulties in the development of most bispecific antibody formats. The Fab format antibody offers advantages over whole IgG, including simple production of the antibody using inexpensive prokaryotic expression systems and better penetration into tumor tissues.^36^ Comparing with single-chain Fv, single-domain antibodies have better biophysical properties, such as less aggregation, high expression levels in *E. coli*, high solubility, and stability *in vitro* compared with scFvs.^37^, ^38^ Based on these properties of single-domain antibodies, we designed several bispecific antibodies by linking a VHH to a native Fab fragment.^13^, ^22^, ^24^, ^39^ These antibodies, including Bp-Bs in this study, can be expressed in *E. coli* with potent tumor-killing properties.

In previous studies, the combination of trastuzumab with pertuzumab was a more effective therapy than mono-therapies in the clinic.^40^, ^41^, ^42^ Moreover, a triparatopic tribody, consisting of three non-overlapping ErbB2 binders, was efficient in downregulating ErbB2 and inhibiting tumor cell growth on Her2-positive tumor cells, including trastuzumab-resistant cell lines.^29^ These studies suggested that targeting different epitopes of Her2 may provide more efficacious therapy for Her2-positive tumors. In this study, we observed that Bp-Bs provided better anti-tumor activities than mono- and bivalent bispecific antibodies targeting Her2. To our knowledge, this is the first report of a biparatopic anti-Her2 bispecific antibody based on the Fab format and anti-Her2 VHH. The potent *in vitro* and *in vivo* activities of Bp-Bs support its advance into clinic for Her2-positive tumors.

## Materials and Methods

### Cell Lines and Animals

The human breast cancer cell lines MCF7, SKOV3, and SKBR3; the colon cancer cell line LS174T; and the Chinese hamster ovary cell line CHO were obtained from the Type Culture Collection of the Chinese Academy of Sciences (Shanghai, China). MCF7, SKBR-3, and SKOV3 cells were cultured in DMEM (Gibco, Thermo Fisher Scientific, Waltham, MA, USA) with 10% heat-inactivated fetal bovine serum (HI-FBS; Gibco, Thermo Fisher Scientific) and 1% penicillin/streptomycin. LS174T and CHO cells were cultured in RPMI-1640 medium (Gibco, Thermo Fisher Scientific) with 10% HI-FBS (Gibco, Thermo Fisher Scientific) and 1% penicillin/streptomycin. All cells were incubated at 37°C in a humidified incubator with 5% CO_2_.

Female NOD/SCID mice and female CB-17 SCID mice were purchased from Vital River Laboratory Animal Technology (Beijing, China). The animals were acclimated for 1 week prior to experiment. The mice were housed in a temperature- and humidity-controlled environment with a controlled light-dark cycle (12-12 h). Animal care and experimental procedures were approved by the Institutional Animal Care and Use Committee, Sun Yat-Sen University.

### Construction, Expression, and Purification

The diagrams of Bi-Bs and Bp-Bs are shown in Figures 1A and 1B. DNA-shuffling and ligation techniques were used to clone the respective genes. Briefly, for Bi-Bs, the single domain anti-Her2 VHH1^43^ was linked to the C termini of the VH-CH1 (with linker: (GGGGS)_3_) and VL-CL of the anti-CD3 UCHT1 clone.^13^ While for Bp-Bs, another anti-Her2 VHH2^12^ was used to replace the previous anti-Her2 VHH1 at the VH-CH1 of the Bi-Bs. The resultant heavy-chain and light-chain genes were then respectively cloned into the pET26b vector (heavy chain [HC]) and pET21a vector (light chain [LC]).

The bispecific antibodies were expressed using the M9 minimal medium method, as described previously.^26^, ^27^ Briefly, the expression vectors HC and LC were co-transfected into *E. coli* BL21(DE3) cells and cultured in Luria-Bertanii (LB) medium with antibiotics at 37°C. The culture was then transferred to M9 minimal medium (12.8 g/L Na_2_HPO_4_, 3.0 g/L KH_2_PO_4_, 0.5 g/L NaCl, 2.0 g/L NH_4_Cl, 20 g/L glucose, 0.1 mM CaCl_2_, 1.0 mM MgSO_4_, and 10 μM FeCl_3_) and incubated at 37°C and 220 rpm in a rotary shaker. When the cell culture reached an optical density (OD) of ∼2.0, IPTG (final concentration, 1 mM) and Tris-HCl (final concentration, 180 mM) were added to induce protein expression and secretion.

Antibody purification was performed as described previously.^44^ Briefly, cells were removed by centrifugation at 4,000 × *g* for 30 min at 4°C, followed by 20,000 × *g* for 30 min at 4°C. The supernatant was recovered and processed for purification. The antibody was purified from the culture supernatant by Ni-NTA Sepharose (GE Healthcare) affinity chromatography. The purified antibodies were analyzed on 8% SDS-PAGE under reducing conditions and non-reducing conditions, followed by Coomassie brilliant blue staining.

### Flow Cytometry Analysis

Flow cytometry was used to assess the binding of bispecific antibodies on Her2-positive or -negative cells. Different cell lines were cultured and resuspended after trypsinization. Cells were then washed and resuspended in 0.1% BSA in PBS. A total of 100 μL 5 × 10^5^ cells/sample was incubated on ice for 1 h in the absence or presence of antibodies. After washing twice with ice-cold PBS, the cells were incubated on ice for 1 h with goat-anti-human IgG (H+L)-AF488 (Invitrogen, A11013). Cell-associated fluorescence was analyzed with a Cytomics FC500 Flow Cytometer (Beckman Coulter Genomics) and plotted using FlowJo (https://www.flowjo.com).

### Immunofluorescence Assay

To further analyze the binding of the antibodies to Her2 on the cell surface, immunofluorescence assay was performed as described previously.^23^ Briefly, the CHO and SKBR3 cells were cultured on the glass-bottom dish (Cellvis) overnight. After washing with PBS three times, the cells were fixed by 4% paraformaldehyde. After blocking with PBS plus 1% BSA for 1 h at room temperature, the cells were incubated with antibodies for 1 h at room temperature. After washing three times with PBS, the samples were incubated with the goat anti-human IgG (H+L)-AF488 for 1 h at 4°C. After washing with PBS, samples were examined using confocal laser-scanning microscopy (Zeiss EC Plan-Neofluar 40×/1.30 Oil DIC M27 objective) and analyzed by ZEN software.

### Affinity Measurement

The affinity of anti-Her2 antibodies to the extracellular region of Her2 protein was determined using an OctetQKe instrument (Pall). Briefly, human Her2 with Fc tag (AcroBiosystem, HE2-H5253) in phosphate buffered saline with Tween 20 (PBST) was loaded onto the surface of ProteinA Capture Biosensors (ProA). Immobilization levels between 0.8 and 1.2 nM were reached. Then a 60-s biosensor baseline step was used before the analysis of association of the antigen and antibodies on the biosensor to the testing antibodies and antigen for 180 s. Tested molecules were then applied in a 2-fold concentration gradient. Octet data were evaluated using data analysis software version 8.2 (PALL/ForteBio), and a global fit 1:1 modal was used to determine the diffusion constant (K_d_) value.

### Antibodies Induce T Cell-Mediated Cytotoxicity

To measure the cytotoxicity of bispecific antibodies *in vitro*, human PBMCs were freshly prepared from fresh donated blood using a gradient centrifugation method with Ficoll-Plaque Plus (GE Healthcare). Human CD3^+^ T cells were then isolated from the PBMCs using an EasySep Human CD3 Positive Selection kit (STEMCELL Technologies, Vancouver, BC, Canada), according to the manufacturer’s instructions. Cytotoxicity assays were performed as described previously.^39^ Briefly, SKOV3, MCF7, LS174T, or CHO cancer cells were trypsinized and seeded at a density of 5,000 cells/well in 96-well tissue culture plates as target cells and incubated overnight at 37°C in 5% CO_2_. A total of 50,000 human CD3^+^ T cells/well without prior stimulation was then added as effector cells. Different concentrations of anti-Her2 antibodies were added to distinct wells. After 72 h of incubation, the Cell Counting Kit-8 reagent (Dojindo, CK04) was applied to quantify cell viability, according to the manufacturer’s protocol. The survival rate (%) of the target cells was calculated using the following formula: [(live target cells (sample) − medium)/(live target cells (control) − medium)] × 100%.

### Immunoblot Analysis of Cell Lysates

SKOV3, LS174T, or MCF7 cells were seeded (300,000 cells/well) in 6-well plates and incubated at 37°C overnight. The cells were then treated with or without 100 nM anti-Her2 antibodies for 30 h at 37°C. After incubation, the cells were washed twice with cold PBS and lysed using radioimmunoprecipitation assay (RIPA) lysis buffer (Beyotime, P0013B), according to the manufacturer’s instructions. Protein concentration was determined by the BCA method (Thermo Fisher Scientific), and protein samples of 20 μg each were analyzed by 8% SDS-PAGE and immunoblotted with antibodies against ErbB2, phospho-ErbB2-Tyr1221/1222, AKT, phospho-AKT-Ser473, p44/42 MAPK, phospho-p44/42 MAPK-Thr202/Tyr204, and tubulin (Cell Signaling Technology, 4290, 2243, 4691, 4060, 4695, 9101, and 2144, respectively).

### Pharmacokinetic Study

Single-dose pharmacokinetic (PK) study of Bi-Bs and Bp-Bs was performed in female CB-17 SCID mice. The animals were randomized into different treatment groups (n = 9 per group, 3 animals per time point), and they were injected intravenously with 1 mg/kg Bi-Bs or Bp-Bs. The serum samples were collected at 0.25, 0.5, 1, 2, 4, 8, 12, 24, and 48 h post-injection for bioanalytical measurement. Antibody concentrations in serum were determined by the ELISA method, as described previously.^45^ PK analyses were performed according to standard non-compartmental analysis using Kinetica (version [v.]5.1 SP1, Thermo Fisher Scientific).

### *In Vivo* Therapy Study

For the *in vivo* xenograft studies, LS174T human colon carcinoma cells were harvested from the cell culture, washed twice with PBS, and then resuspended in PBS. A total volume of 200 μL/mouse containing 1 × 10^6^ LS174T cells was injected subcutaneously into the right flank of NOD/SCID mice. When the tumor size reached 50–100 mm^3^, 5 × 10^6^ fresh isolated human PBMCs were administered intraperitoneally. Then animals were treated with different dosages of antibodies or control vehicles. Mice were weighed, and the tumor volume was determined in two perpendicular dimensions and calculated using the following formula: (length × width^2^)/2. Mice were sacrificed when the tumor volume reached 1,500 mm^3^. All the results are presented as the arithmetic mean for each group.

### Statistical Analysis

All statistical analyses were performed using GraphPad Prism 7.0 software (GraphPad, La Jolla, CA, USA). Statistical analysis was performed with ANOVA followed by Dunnett’s multiple comparisons test unless otherwise noted. A non-linear regression analysis was used in Figure 3B. p < 0.05 indicated a statistically significant difference. Data were described as the mean ± SEM unless otherwise noted.

## Author Contributions

Z.W. designed the study strategy. J.L., X.W., L.L., H.P., Y.W., Y.L., and Y.Z. performed experiments. J.L. and L.L. performed analysis. Z.W. and J.L. prepared the manuscript.

## Conflicts of Interest

The authors declare no competing interests.
